# Surface Characterization of N‐Heterocyclic Carbenes on Gold and Silver: Exploring Distinct Crystalline Facets

**DOI:** 10.1002/cphc.202500227

**Published:** 2025-07-02

**Authors:** Sayantan Mahapatra, Linfei Li, Nan Jiang

**Affiliations:** ^1^ Department of Chemistry University of Illinois Chicago Chicago Illinois 60607 USA; ^2^ Department of Physics University of Illinois Chicago Chicago Illinois 60607 USA

**Keywords:** nanoelectronics, N‐heterocyclic carbene, scanning tunneling microscopy, single‐molecule imaging, ultrahigh vacuum

## Abstract

Due to its exceptional chemical and functional versatility, N‐heterocyclic carbene chemistry holds great promise in replacing thiol chemistry for applications in next‐generation nanoelectronics. To manipulate their chemical properties and interfacial characteristics, a wide range of substrates needs to be employed for the formation of self‐assemblies. Research has, however, predominantly focused on the Au(111) substrate. Herein, a systematic approach is presented for producing long‐range, densely packed self‐assemblies using carbenes deposited over (111) and (100) crystalline facets of gold and silver single crystals. Atomic‐resolution characterization with scanning tunneling microscopy (performed at 78 K and room temperature) is combined with the molecular structure, showing the role of distinct crystal facets in the ordering of self‐assembled monolayers.

## Graphical abstract

This study investigates the self‐assembly of N‐heterocyclic carbenes (NHCs) on gold and silver substrates with different crystalline facets. A marked difference in assembly is observed when transitioning from (111) to (100) facets. Additionally, surface reconstruction appears to significantly influence the assembly behavior. The work offers plausible explanations for the facet‐dependent differences in molecular organization.

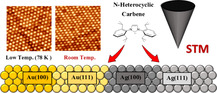

## 1 Introduction

Compact self‐assembled monolayers (SAMs), consisting of well‐defined upright‐oriented molecular films over vast varieties of solid substrates, are considered one of the most interesting systems in terms of wide varieties of nanotechnological applications such as molecular electronic devices, molecular diodes, switches, organic field‐effect transistors (OFETs), organic photovoltaic devices (OPVs), sensors, and so forth.^[^ ^1^ ^]^ Owing to the many different aspects of the surface superstructure (such as different crystal facets) and molecular design, the assembly process is controlled by energetically favorable interactions such as van der Walls forces, electrostatic forces, and hydrogen bonding.^[^ ^2^ ^]^ The ultimate assembly process and the performance of modern‐day electronic devices are determined by the careful balance between all these competing forces.^[^ ^3^ ^]^ However, fabrication of these real‐life molecular electronic devices still requires the optimization of two fundamental parameters: first, the formation of highly ordered, densely packed (defect‐free) SAMs over a different range of solid substrates, and second, the robustness and compatibility at room temperature (RT).^[^ ^4^ ^]^ The presence of defects, either from the different crystal facets or from the adsorbed molecules (such as molecular disorder, domain boundaries, and polymorphism), has a strong impact on molecular and surface engineering, thereby prohibiting the successful design of the devices. Indeed, intermolecular coupling (i.e., ordering) is of key importance for efficient lateral transport in OFET based on SAMs (SAMFETs). On the other hand, these stable, low‐defective molecular structures should provide thermal stability for daily‐life electronic applications. Therefore, any SAM‐related nanotechnological application demands densely packed, highly ordered, strongly bonded (chemisorbed), and thermally stable SAMs over different sets of solid substrates.

In the last three decades, SAMs involving thiol functional groups (sulfur‐containing molecules such as alkanethiols; RS—H) on gold (Au) substrates have been extensively studied using both experiment and theory where sulfur binds with the Au atom (S—Au).^[^ ^5^ ^]^ However, this type of thiol‐based SAMs suffers from relatively low thermal stability, polymorphism, and in some cases, the molecular chemistry remains a matter of controversy.^[^ ^6^ ^]^ For example, the process of thiol end‐groups (S—H) binding to Au (S—Au) generally assumes the dissociation and removal of hydrogen atom, but recent evidence suggests otherwise.^[^ ^7^ ^]^ Therefore, in a quest for a more sustainable candidate, N‐heterocyclic carbenes (NHCs) have been proposed as an extremely interesting replacement to generate stable SAMs over Au substrate via C—Au chemical bond.^[^ ^8^ ^]^ Recent studies suggest, due to the higher stability of the C—Au bond over S—Au, NHC‐based SAMs can experience significantly higher thermal and chemical stability compared to the thiol counterparts.^[^ ^9^ ^]^ Furthermore, they form thin insulating monolayers with significantly high quality and stability. However, in addition to their comparatively high chemical stability, highly ordered, densely packed, 2D NHC‐based SAMs (low‐defect concentrations) over vast varieties of solid substrates are of immense importance for suitable nanoelectronic applications. For instance, high thermal and chemical stabilities have been observed for 2D disordered NHC structures on Au(111). On the other hand, 2D ordered structures have been realized for flat‐lying NHCs (as configuration depends strongly on the ligands attached to NHCs) structures with much less thermal stability compared to standing‐up NHC monolayers.^[^ ^10^ ^]^

**Figure 1:**
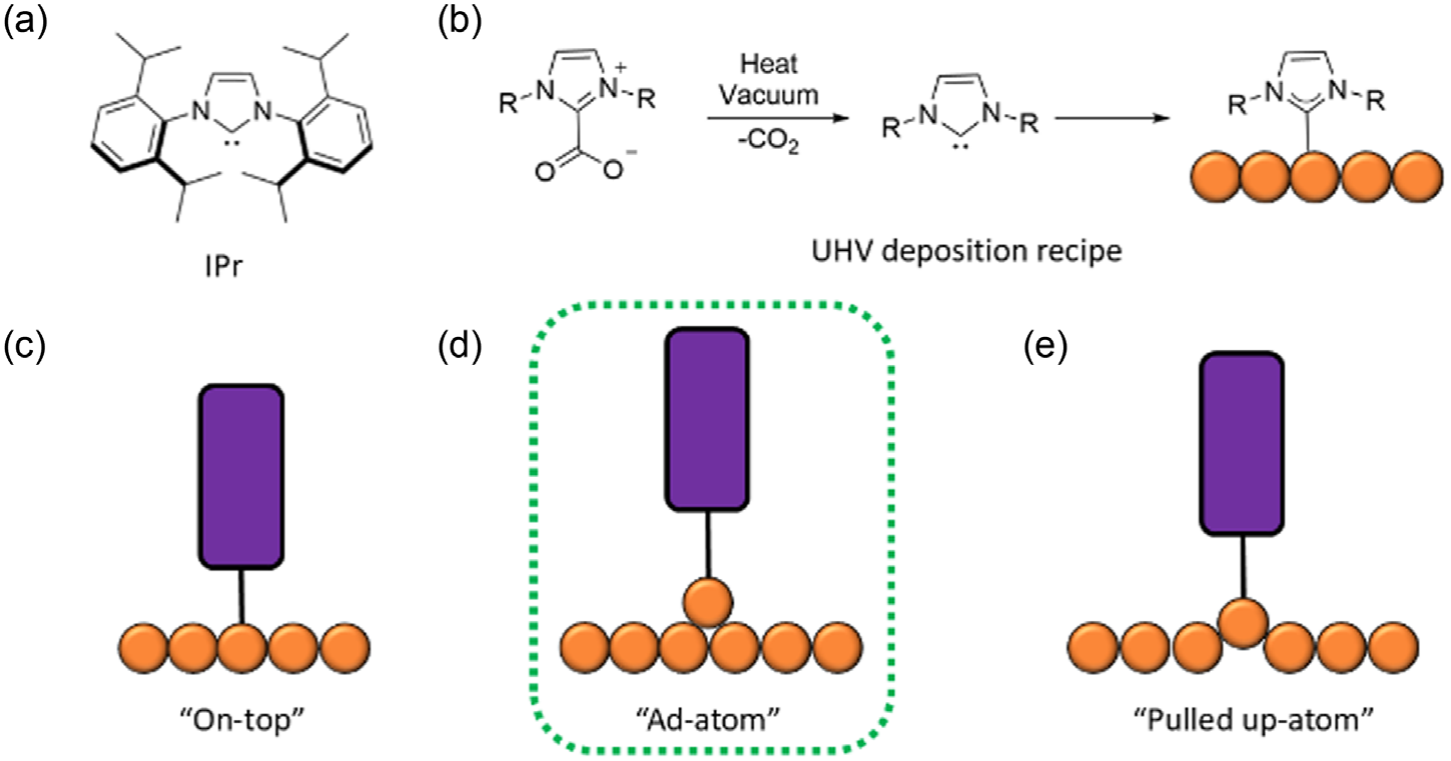
a) Chemical structure of IPr. b) Ultrahigh vacuum deposition recipe of NHCs on metal surfaces. Illustration of c) “on‐top”, d) “ad‐atom”, and e) “pulled up‐atom” configurations of standing‐up NHCs on surfaces.

In this work, we aimed to develop an NHC model to study and demonstrate the formation of SAMs over different sets of substrates that exhibit long‐range 2D ordering and low defect concentration in a standing‐up (upright‐oriented) configuration. NHC‐based SAMs on Au(111) are by far the most studied, in which highly ordered NHC structures were found recently.^[^ ^11^ ^]^ Recent reports also explored the adsorption behavior over copper, silicon, and 2D metal (borophene) surface.^[^ ^12^ ^]^ However, in comparison to Au(111), NHC‐based SAMs remain largely unexplored over Au(100) surface facet, even if, both the (111) and (100) planes are stable crystalline phases in Au nanoparticles (AuNPs) and their distribution is controlled by particle size which is immensely important in biomedical applications.^[^ ^13^ ^]^ Low‐index planes like the (111) and (100) facets of gold are commonly used as substrates for molecular nanostructures due to their relative chemical inertness and well‐defined, long‐range surface reconstructions.^[^ ^14^ ^]^ These reconstructions result from significant rearrangements of surface atoms, leading to distinct surface features—for example, the fcc/hcp stacking in (111) and the steeper versus flatter ridges in (100).^[^ ^14^ ^]^ Consequently, for the growth of ordered structures, the local structural variations inherent in the reconstructions of (111) and (100) facets are crucial factors. In our experiments, we chose NHC SAMs based on 1,3‐bis(2,6‐diisopropylphenyl)imidazolium‐2‐carboxylate, referred to as IPr–CO_2_ (**Figure** **1**a). This particular NHC has been shown to successfully adopt a standing‐up configuration on Au(111), as demonstrated in prior studies utilizing both density functional theory (DFT), high‐resolution X‐ray photoelectron spectroscopy (HR‐XPS), and near‐edge X‐ray absorption fine‐structure spectroscopy (NEXAFS).^[^ ^15^ ^]^ Moreover, according to previous studies, upright (standing‐up) carbene molecules are proposed to adopt at least three distinct configurations: i) “on‐top” bonding directly to a surface atom (Figure 1c); ii) bonding to an Au ad‐atom, referred to as the “ad‐atom” configuration (Figure 1d); and iii) bonding to a “pulled‐up” atom from the surface (Figure 1e).^[4a]^ In our case, after deposition to the metal surface, the IPr molecules tend to adopt “ad‐atom” configuration (Figure 1d), as suggested by recent experimental and theoretical reports.[^11^, ^15^] The atomic‐scale analysis of individual molecules and large‐scale ordered superstructures on these distinct substrate facets [i.e., Au(100) and Au(111)] was performed using high‐resolution scanning tunneling microscopy at 78 K and RT. Experiments were conducted on two unreconstructed silver (Ag) facets, specifically Ag(100) and Ag(111), to assess their viability across different noble metal surfaces. Our findings demonstrate that a minor alteration in the surface facet effectively removes molecular disorder, resulting in well‐ordered SAMs with similar surface coverages. Additionally, the thermal stability of these long‐range, ordered structures was confirmed through room‐temperature STM imaging, indicating their potential compatibility with next‐generation electronics under real‐world conditions.

## 2 Results and Discussion

### 2.1 IPr on Au(100)

**Figure 2:**
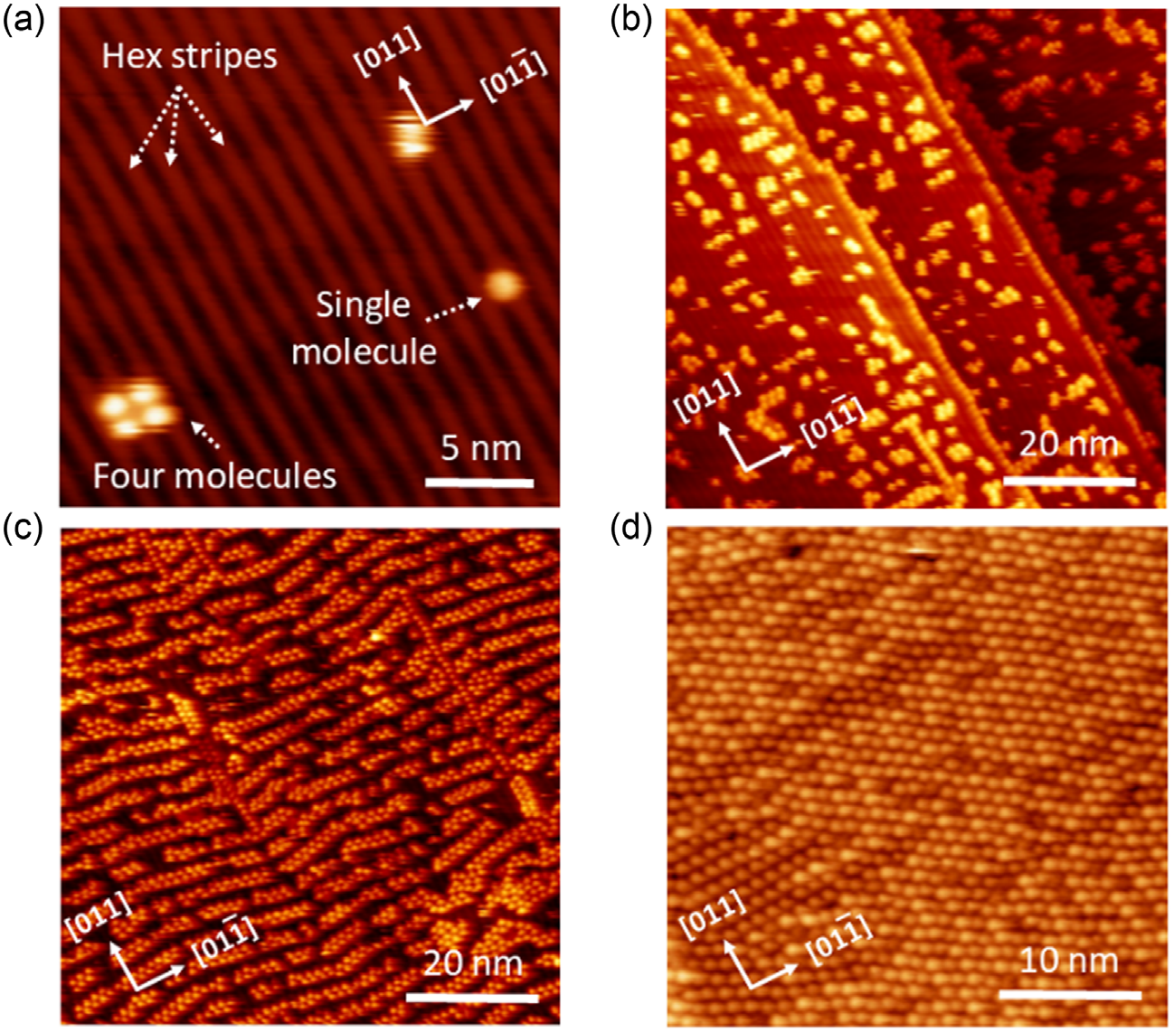
a) Constant‐current STM topography of IPr deposition on Au(100) surface at very low molecular coverage. Hex stripes, a molecular cluster, and a single molecule are identified. Corresponding STM images at b) about half‐monolayer, c) close to full monolayer, and d) saturation coverage. STM conditions (78 K): (a) −2.75 V, 20 pA. (b) −2.50 V, 15 pA. (c) −2.66 V, 20 pA. (d) −2.84 V, 15 pA.

**Figure 3:**
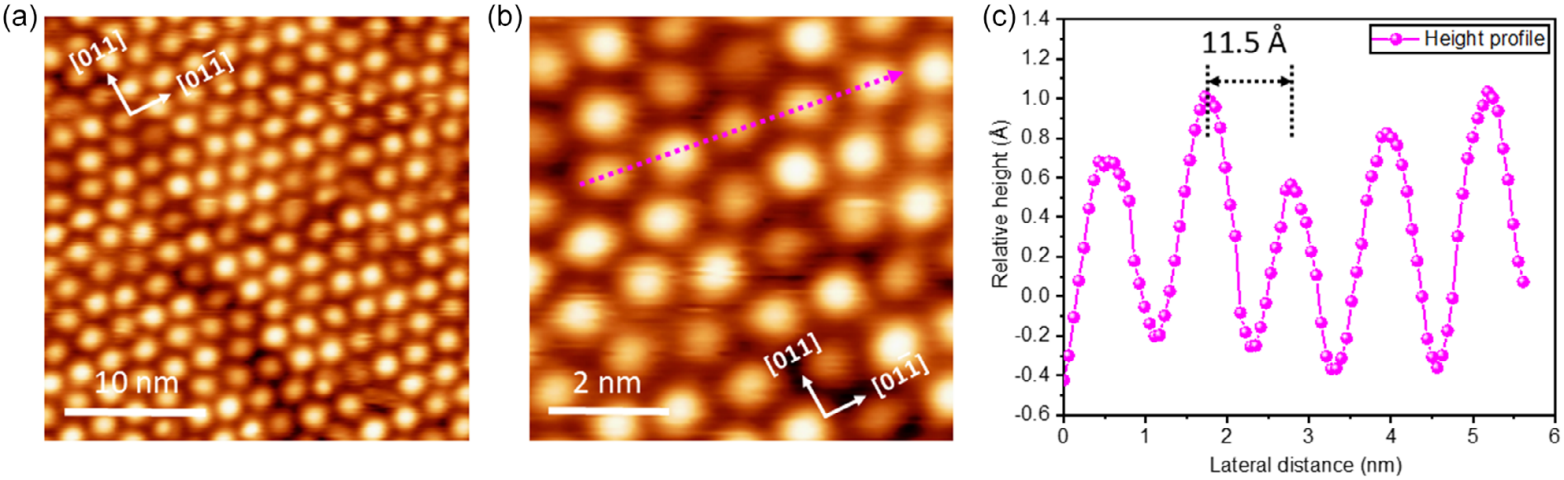
a) Large‐scale STM image of IPr decoration on Au(100) surface at the saturation coverage. b) Zoom‐in STM image of non‐uniform molecular decoration. c) STM height profile of corresponding pink dotted arrow, indicating nonuniform height inside the molecular self‐assembly. STM conditions (78 K): (a,b) −2.75 V, 15 pA.

The IPr molecule chosen for this study is depicted in Figure 1b, which shows its fully upright configuration on a solid substrate. Our experiment starts with the sublimation of the CO_2_ adduct of IPr (IPr–CO_2_) onto the (100) facet of Au under ultrahigh vacuum (UHV) conditions (Figure 1b). This process results in the IPr molecules being attached to the Au surface (sample maintained at RT) through direct C—Au bonding, ensuring a pristine attachment without any impurities.[^11^, ^16^] The quasihexagonal “hex” superstructures of the Au(100) facet are generally more contracted than those of the bulk (111) facet. This extensive surface “hex” reconstruction results in the formation of steeper and flatter ridges in the surface superstructure, offering varied landing sites on the terraces for IPr molecules during nanostructure growth. At low molecular coverage, IPr molecules predominantly adsorb along the step edges. A few scattered single molecules and clusters consisting of 3–4 molecules were observed on the “hex” reconstructed terraces, which occur only after the step‐edges have been fully saturated by the molecules, as depicted in **Figure** **2**a. The round‐shaped appearance of the molecules in Figure 2a is attributed to the free rotation around the single bond between the IPr carbon atom and the Au atom.^[11a]^ The STM line profile indicates varying heights for the individual molecules, suggesting that they may settle on either the steeper or flatter ridges of the surface (Figure S1, Supporting Information). Even when the coverage was increased to approximately half a monolayer (Figure 2b), no long‐range ordered structures were observed, indicating a relatively strong molecule‐substrate interaction on this facet. However, the IPr molecules were observed to form either linear chain‐like structures, where two molecules align side by side, or small, disordered clusters consisting of 4–6 molecules. The linear growth of the molecular pairs tends to orient nearly perpendicular to the “hex” reconstructed rows. As the coverage approached a full monolayer, the prevalence of these linear chain structures increased significantly, as illustrated in Figure 2c. The scratchiness observed in the images suggests that the molecules are prone to displacement during scanning under the current experimental conditions (78 K). At saturation coverage (full monolayer), the molecules formed a long‐range, well‐ordered hexagonal‐close‐packed (*hcp*) structure, as shown in Figure 2d and 3a. Interestingly, STM imaging remains highly stable at full‐monolayer coverage, as the IPr molecules are firmly held in place by interactions with neighboring molecules. This strong molecular arrangement results in a well‐structured and stable SAM with pronounced IPr–IPr interactions (**Figure** **3**a,b). Inside the *hcp* structure, the distance between the IPr molecules is measured to be 11.5 ± 0.2 Å (Figure 3c). Several notable observations can be made: First, at saturation coverage, the STM image (Figure 3a,b) shows the absence of the previously observed “hex” reconstructed rows, indicating significant morphological changes in the hex stripes upon reaching saturation. Second, within the molecularly ordered structure, there is a long‐range “nonuniformity,” as evidenced by the presence of various distinct bright features, which is further highlighted by the height profile in Figure 3c.

### 2.2 IPr on Au(111)

**Figure 4:**
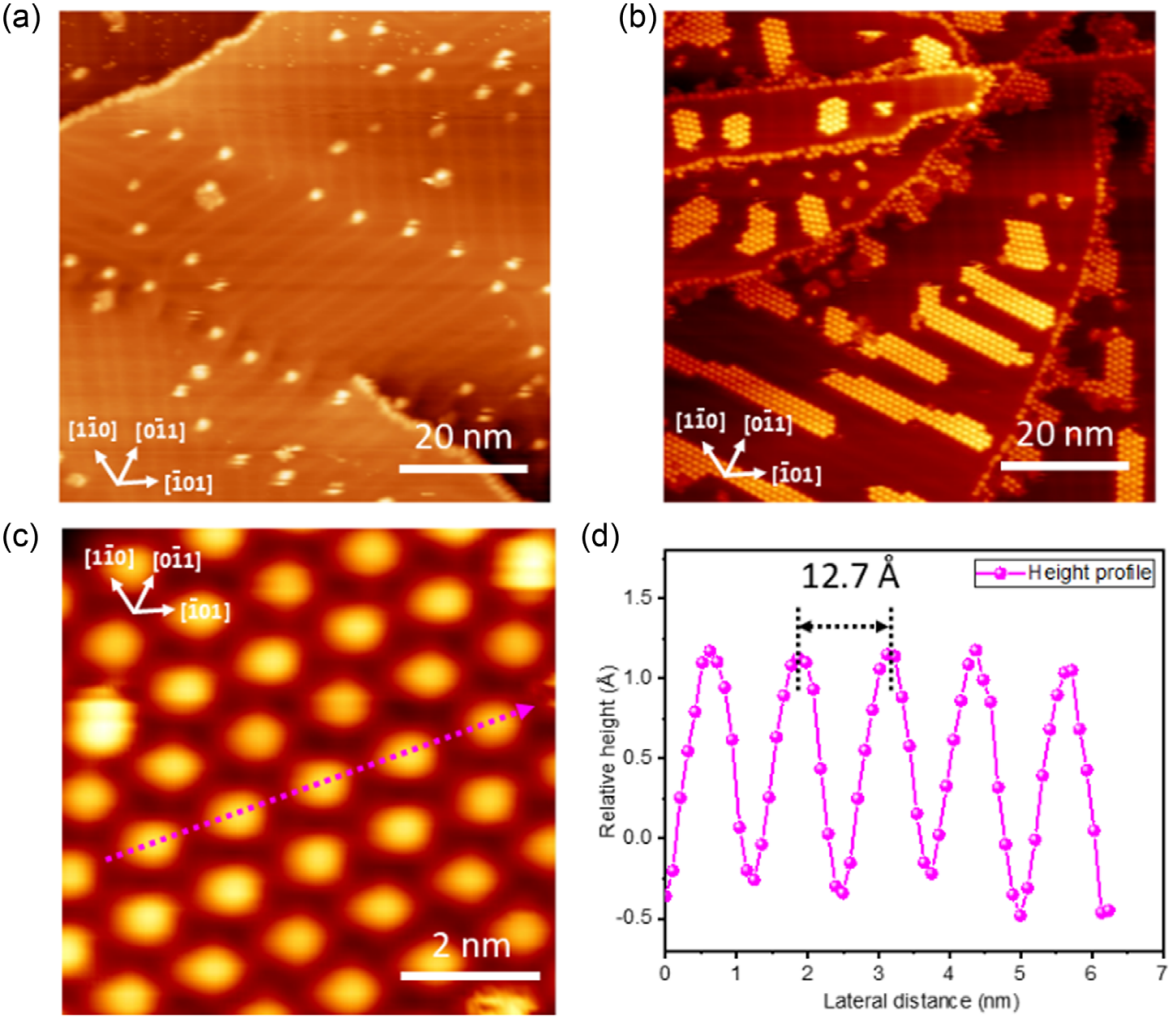
a) Constant‐current STM topograph of IPr deposition on Au(111) surface at very low molecular coverage. b) Corresponding STM image at submonolayer coverage, showing multiple self‐assembled molecular islands. c) Zoom‐in STM image of IPr molecules inside the molecular islands. d) STM height profile of corresponding pink dotted arrow. STM conditions (78 K): (a) −2.84 V, 20 pA. (b) −2.84 V, 15 pA. (c) −2.80 V, 15 pA.

To compare our findings, we deposited the IPr molecules on the (111) facet of Au, where long‐range fcc/hcp ordered stacking patterns, commonly referred to as “herringbone” reconstruction, are observed. At very low surface coverage, the molecules predominantly appear along the step edges and elbow positions of the “herringbone” reconstructed Au surface (**Figure** **4**a). In stark contrast to the results obtained from the Au(100) surface, ordered IPr molecular islands can form at submonolayer coverage on the Au(111) surface. These islands preferentially occupy the fcc stacking regions (Figure 4b) and exhibit a similar hexagonal‐close‐packed structure (Figure 4c). The distance between the IPr molecules is measured to be 12.7 ± 0.4 Å, as shown in Figure 4d. Under experimental conditions (78 K), the molecular islands were easily disrupted or fragmented into smaller pieces, reflecting the high mobility of the IPr molecules. These observations align well with the previous report.^[11a]^ We examined the formation of defects in this sample, as this remains a crucial aspect for nanoelectronic applications. Tip‐induced defects readily appeared during the scanning of the same area under this condition (Figure S2, Supporting Information), suggesting a relatively weak C—Au bond in this system. In contrast, we did not observe tip‐induced defect formation on the (100) facet at the saturation coverage. These findings indicate that it is possible to minimize molecular disorder and create large, ordered molecular domains in an NHC‐based network by switching the crystal facets from (100) to (111) on the Au surface. Subtle variations in molecule‐substrate interactions between these two facets significantly impact our ability to manipulate molecular properties, which is crucial for constructing nanoscale devices.

### 2.3 IPr on Ag(100) and Ag(111)

**Figure 5:**
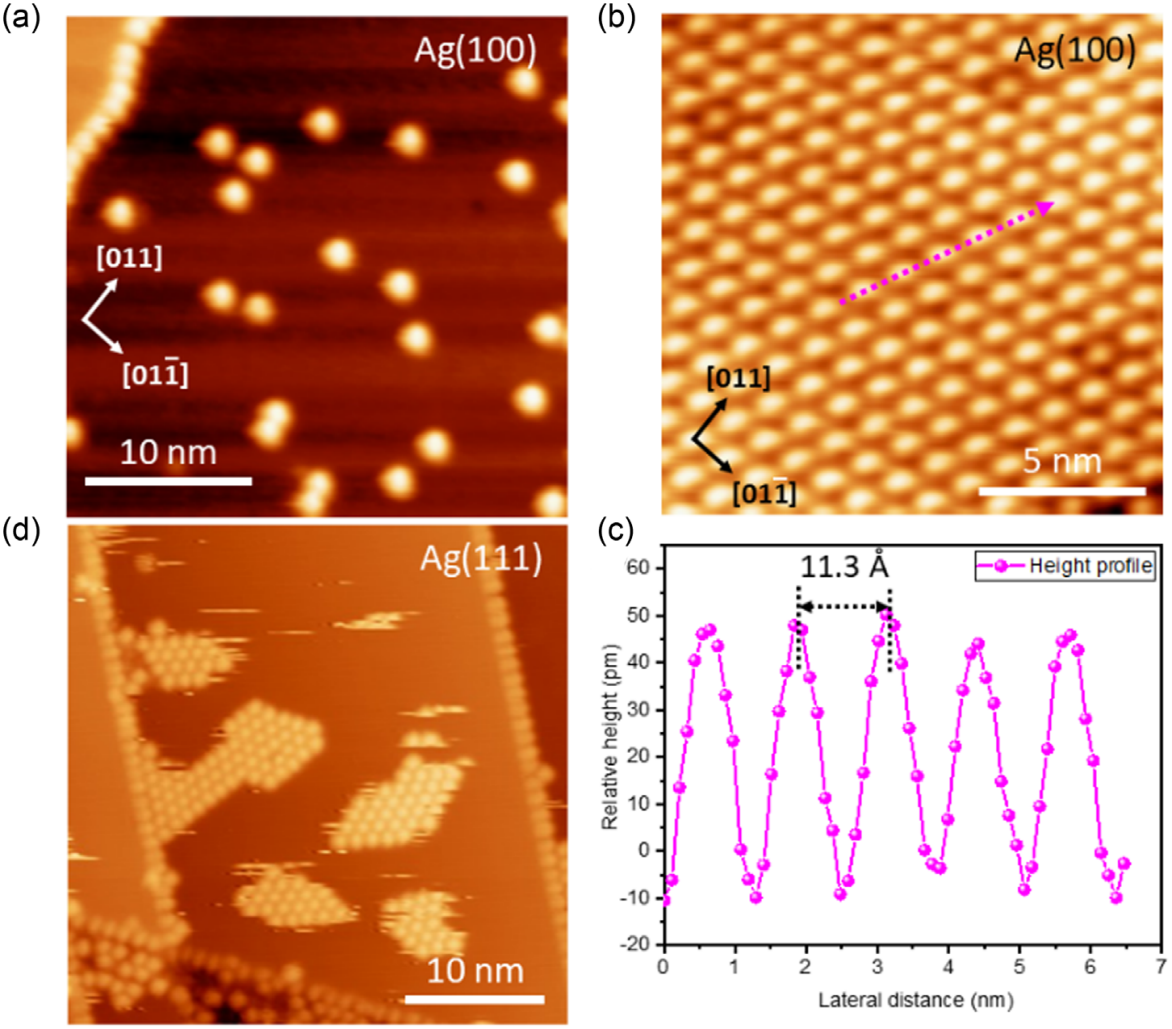
a) Constant‐current STM topograph of IPr on Ag(100) surface at submonolayer coverage, identifying single molecules. b) STM image at saturation coverage. c) STM height profile along the pink dotted arrow, showing uniform molecular decoration. d) STM topograph of IPr deposition on Ag(111) surface at submonolayer coverage, identifying unstable molecular islands. STM conditions (78 K): (a) −2.84 V, 30 pA. (b) −2.84 V, 20 pA. (c) −1.0 V, 50 pA.

To gain a broader perspective, we now focus on the same crystalline facets of the Ag surface, specifically Ag(100) and Ag(111), where no such surface reconstructions are observed. As observed for the Au(100) sample, the round‐shaped bright protrusions scattered across the surface are attributed to single chemisorbed IPr molecules on the Ag(100) surface at submonolayer coverage, with no visible ordered molecular domains (**Figure** **5**a). As indicated by previous DFT studies, NHCs exhibit similar behavior on both Au and Ag surfaces, so we anticipate a comparable adsorption geometry (i.e., “ad‐atom” configuration) in this case.^[^ ^17^ ^]^ Single molecules appear at a similar height (Figure S3, Supporting Information), which suggests a chemically uniform binding state on the Ag(100) surface. At full‐monolayer coverage, a compact, defect‐free hexagonal close‐packed structure was observed, as depicted in Figure 5b. Interestingly, the molecular arrangement appears highly uniform, with all molecules exhibiting a round shape and consistent topological features across the domain, as shown by the height profile (Figure 5c). This is in contrast to the results observed for the Au(100) crystal. Thus, the observed “nonuniformity” in the *hcp* network on Au(100) likely arises from the long‐range “hex” surface reconstruction at the top layer. This will be discussed in more detail later. Finally, these molecules are deposited on the Ag(111) surface. As with their behavior on Au(111), ordered molecular islands were observed at the submonolayer coverage (Figure 5d). These islands are unstable and can be easily displaced during STM imaging under these conditions (Figure S5, Supporting Information).

For practical electronic applications, robustness and compatibility at room temperature are essential. To address this, we conducted room‐temperature molecular‐resolution characterization of IPr SAMs at saturation coverage on Au(100) and Ag(100) surfaces. The long‐range ordering and nano‐patterning of the SAMs were maintained at room temperature, with the hexagonal‐close‐packed structures exhibiting both “nonuniform” and “uniform” patterns on Au(100) and Ag(100) surface, respectively (Supporting Information, Figure S4). This demonstrates the stability of SAMs at room temperature on noble metal surfaces, highlighting their potential for use in NHC carbene‐based molecular electronics and sensing applications.

### 2.4 Comparative Analysis of IPr on Different Facets

To better illustrate how the same IPr carbene molecules behave depending on the facet orientation of single‐crystal surfaces, we provide a side‐by‐side comparative discussion. First, on the (111) facet, IPr molecules tend to form self‐organized assemblies at submonolayer coverage (room temperature deposition), In contrast, for the same coverage and deposion conditions, they appear as randomly scattered single molecules on the (100) facet. This behavior is consistently observed on both Au and Ag metal surfaces. We would also like to note that the IPr molecules were shown to generate dispersed arrangement (single molecules, dimers, trimers) on Cu(111) surface, whereas on Cu(100), the IPr deposition led to self‐assemblies (nano‐islands).^[^ ^18^ ^]^ Our observations on the behavior of IPr molecules on different crystal facets of Au and Ag surfaces are in good agreement with their behavior on different facets of Cu surfaces.^[^ ^18^ ^]^

Second, at full‐monolayer coverage, we also observe a certain “non‐uniformity” in the structure at the (100) facet of Au surface along with lifting of surface reconstruction. The relative height profile (Figure 3c) suggests a height difference of more than 3 Å inside the compact structure. In contrast, the structure remains relatively uniform at the (100) facet of Ag surface, as shown in Figure 5c. The following section provides plausible explanations for the observed behavior of IPr molecules on different crystalline facets.

### 2.5 Plausible Explanation

The current STM results offer several intriguing insights into NHC‐based SAM chemistry. First, it is noteworthy that IPr molecules can form either disordered or ordered molecular networks with similar submonolayer coverage, depending on the single‐crystal facet. This contrasts with alkanethiols (ATs), which consistently form well‐ordered structures on both facets.^[^ ^19^ ^]^ Understanding the underlying reasons for this discrepancy could provide valuable insights into the behavior of NHC molecules. Second, the presence of certain “nonuniformity” in the long‐range ordered structure of IPr on Au(100) at saturation coverage raises questions about the factors contributing to this effect. Identifying the causes of this nonuniformity is essential for optimizing the molecular assembly and functionality in various applications.

The ability of ad‐molecules to diffuse across the surface along low‐energy barrier path is crucial for the formation of well‐ordered molecular domains. This surface mobility can be influenced by several factors, including the choice of molecules (e.g., size, chemical properties and number of anchoring groups), the underlying substrate (e.g., electronic states near the Fermi level), and different crystal facets. A plausible explanation for the greater ordering observed at the (111) facet could be the differences in the adsorption energetics across the various surface sites. For instance, adsorption at different sites such as the fcc, hcp, and bridge sites for the (111) facet, versus the top, hollow, and bridge sites for the (100) facet, may lead to variations in energy.

Similar findings were reported by Maza et al. for 6‐mercaptopurine (6‐MP) on Au(111) and Au(100) surfaces, where differences in adsorption energies of about 0.2 and 0.5 eV for the (111) and (100) facets, respectively, were noted.^[^ ^20^ ^]^ This difference makes surface diffusion more feasible on the (100) facet. Analogously, we hypothesize that the adsorption of IPr molecules in the upright geometry might show a larger energy difference at the (111) surface sites compared to the (100) facets. At moderate coverage, this variation in adsorption energy on the (100) plane could introduce significant disorder among the IPr molecules. In contrast, the favorable adsorption sites on the (111) facet might facilitate greater mobility of IPr molecules, promoting better ordering and reducing the surface free energy. Additionally, factors such as molecular free rotation and surface‐induced dipole moments could also contribute to the observed enhanced mobility of IPr molecules on the (111) facet compared to that on the (100) facet.

**Figure 6:**
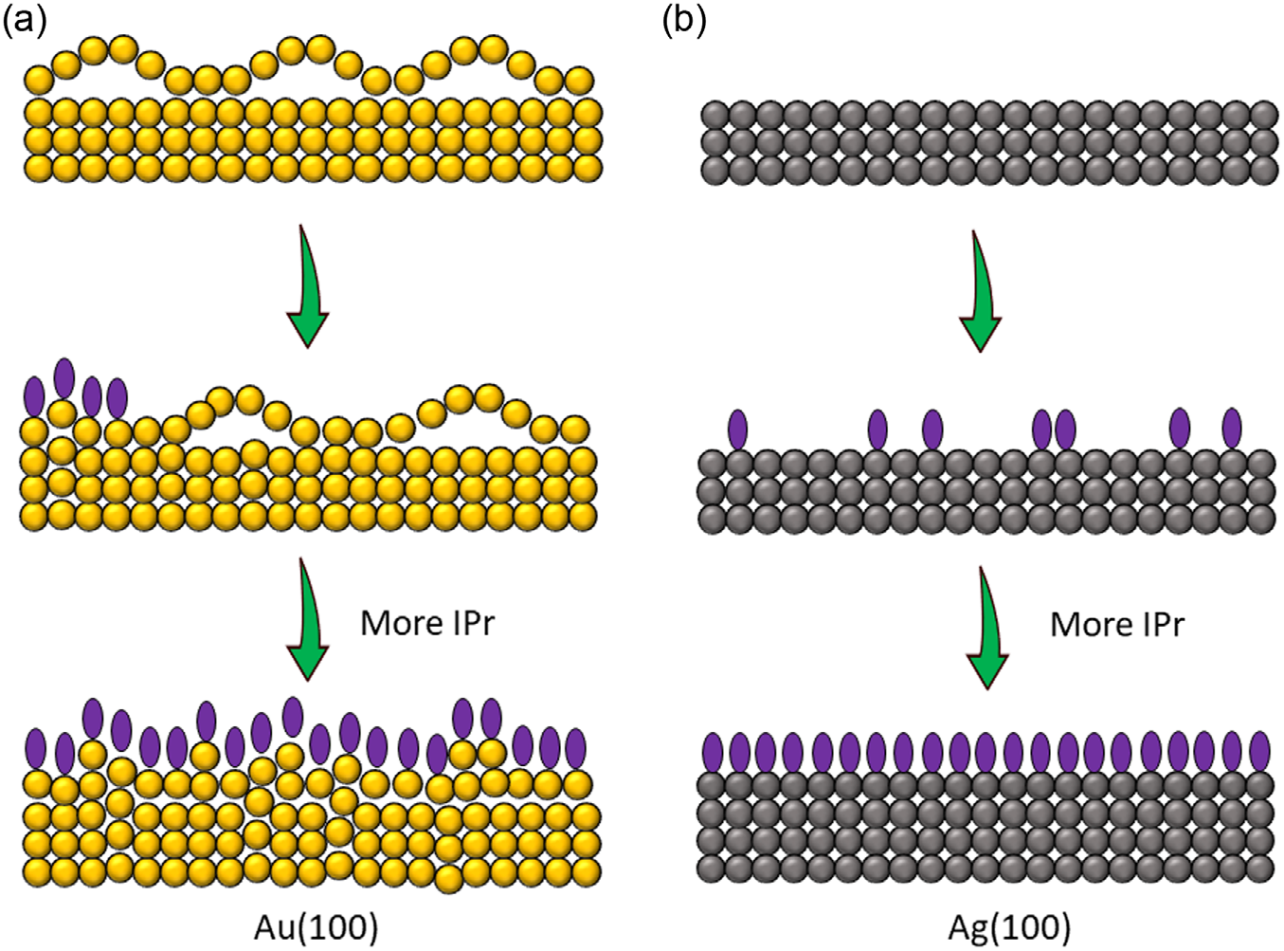
Simplified illustration of “non‐uniform” and “uniform” molecular decoration at saturation IPr coverage over a) Au(100) and b) Ag(100) surface, respectively. The purple ellipsoids represent IPr molecules.

We now address the issue of “nonuniformity” observed on the Au(100) crystal facet. **Figure** **6**a illustrates a simplified model of the Au(100)‐hex reconstructed surface.^[^ ^21^ ^]^ The top layer of this surface exhibits corrugation with steeper and flatter ridges, resulting from periodic misfits between the bulk and the reconstructed lattice. The packing density of the Au(100)‐hex surface is ≈24% more atoms per unit area in the hexagonal layer than in the unreconstructed (1 × 1) surface. Experimental observations confirm that at saturation coverage, the adsorption of IPr molecules disrupts this surface reconstruction, leading to the formation of excess surface Au atoms. In contrast, the (100) facet of the Ag surface does not exhibit such a reconstruction (as shown in the model in Figure 6b), and thus carbenes adopt a uniform “ad‐atom” configuration. This difference suggests that the observed “nonuniformity” in the long‐range ordered structure at saturation coverage on Au(100) is likely due to the disruption of the “hex” reconstruction and the resultant generation of excess surface Au atoms (Figure 6a,b).

## 3 Conclusion

In summary, the exposure of IPr to UHV conditions on different crystal facets, namely (100) and (111) of Au and Ag surfaces, results in either disordered structures [(100) facet] or well‐ordered configurations [(111) facet] for the standing‐up arrangement. Surface characterization revealed both individual molecules and large‐scale ordered structures. At saturation coverage, STM imaging shows that IPr adsorbs in a highly uniform manner on Ag(100), whereas some degree of nonuniformity is observed on the Au(100) surface. This nonuniformity is potentially attributed to the presence of excess Au‐adatoms resulting from the lifting of “hex”‐reconstruction. Furthermore, room‐temperature imaging demonstrated the relevance of these findings to contemporary electronic devices.

## 4 Experimental Section

The experiments were performed using a variable temperature STM system (USM1400, UNISOKU Co., Ltd,) operating under a base pressure of 2 × 10^−10^ torr. The clean surfaces were prepared in a separate preparation chamber with a base pressure of 1 × 10^−10^ torr, which is isolated from the STM chamber by a gate valve. The Au and Ag surfaces were cleaned through repeated cycles of Argon ion sputtering (1 kV, ≈2.5 × 10^−5^ torr) followed by indirect thermal annealing. 1,3‐bis(2,6‐diisopropylphenyl)imidazolium‐2‐carboxylate (IPr–CO_2_) molecules were sublimed on the different surfaces via a K‐cell molecular evaporator (ACME Technology Co., Ltd.). The sample remained at room temperature during the molecule deposition process. Subsequently, it was transferred to the STM chamber for STM imaging experiments conducted at a low temperature of liquid nitrogen (78 K) and room temperature (298 K). Electrochemically etched Ag tips were employed for STM imaging.^[^ ^22^ ^]^

## Conflict of Interest

The authors declare no conflict of interest.

## Supporting information

Supplementary Material

## Data Availability

The data that support the findings of this study are available from the corresponding author upon reasonable request.
