# Supplementary material for: Surface Characterization of N‐Heterocyclic Carbenes on Gold and Silver: Exploring Distinct Crystalline Facets
Source: Chemphyschem. 2025 Jul 2;26(16):e202500227. doi: 10.1002/cphc.202500227 (PMC12388166; doi:10.1002/cphc.202500227)
Supplement: Supplementary file 1 — Supplementary Material [file CPHC-26-e202500227-s001.pdf]

Supporting Information for:

Surface Characterization of N-Heterocyclic Carbenes on Gold and Silver: Exploring Distinct Crystalline Facets

*Sayantan Mahapatra,[1] Linfei Li,[1] and Nan Jiang[1,2] **

[1]Department of Chemistry, University of Illinois Chicago, Chicago, Illinois 60607, United States.

[2]Department of Physics, University of Illinois Chicago, Chicago, Illinois 60607, United States.

Corresponding Author:

*Email: njiang@uic.edu

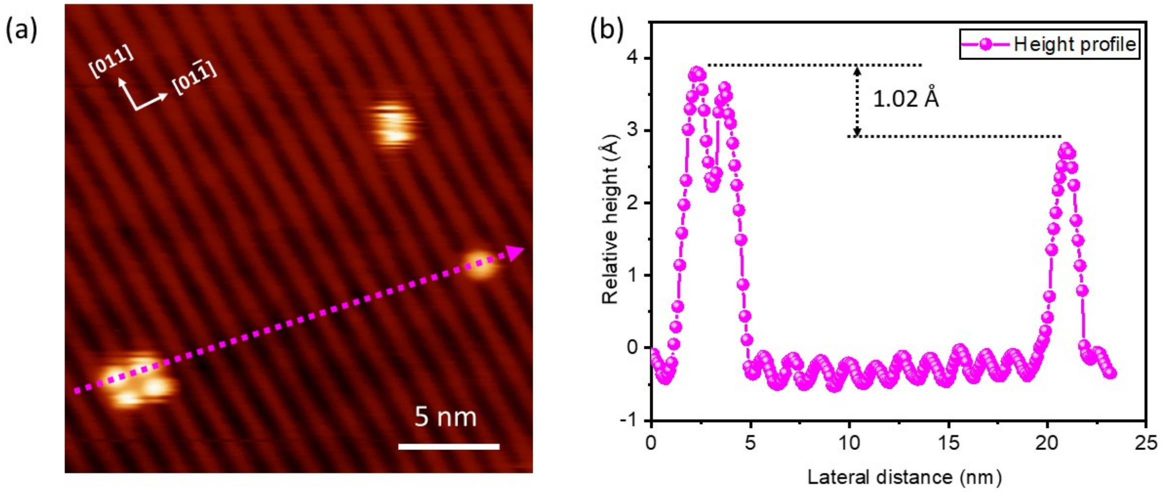

Figure S1. (a) Constant-current STM image of IPr deposition on Au(100) surface at very low coverage. (b) Corresponding STM height profile across the pink dotted arrow, showing the height difference of ~ 1.02 Å. STM condition (78 K): -2.75 V, 20 pA.

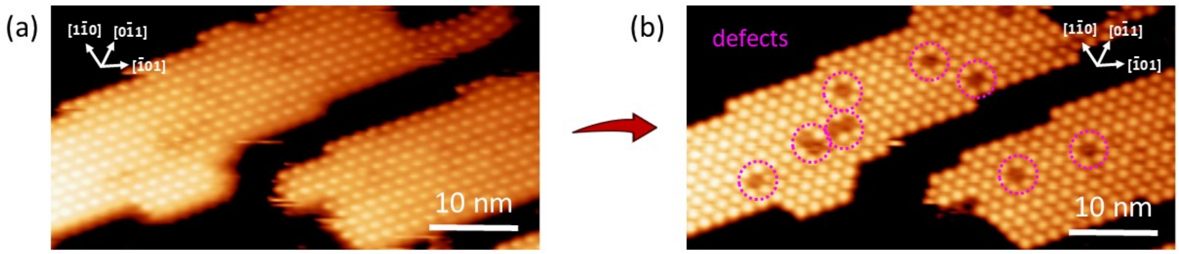

Figure S2. (a) and (b) Tip-induced defects of IPr molecular islands on Au(111) surface. The defects are identified by the pink dotted circles. The defects are formed while scanning. STM conditions (78 K): (a) and (b) -2.80 V, 15 pA.

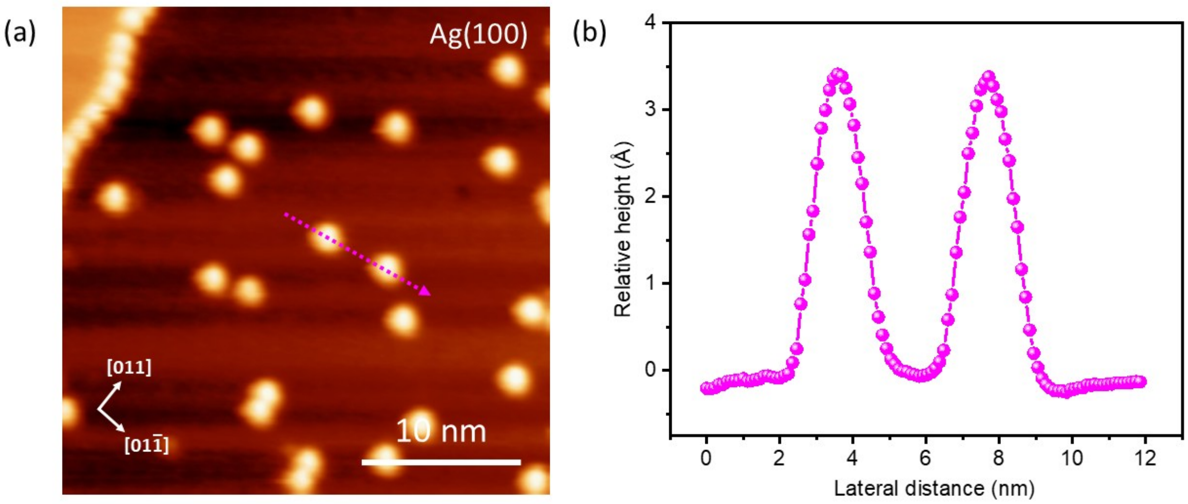

Figure S3. (a) Constant-current STM image of IPr deposition on Ag(100) surface at sub-monolayer coverage. (b) Corresponding STM height profile across the pink dotted arrow, showing similar heights for individual molecules. STM condition (78 K): (a) -2.84 V, 30 pA.

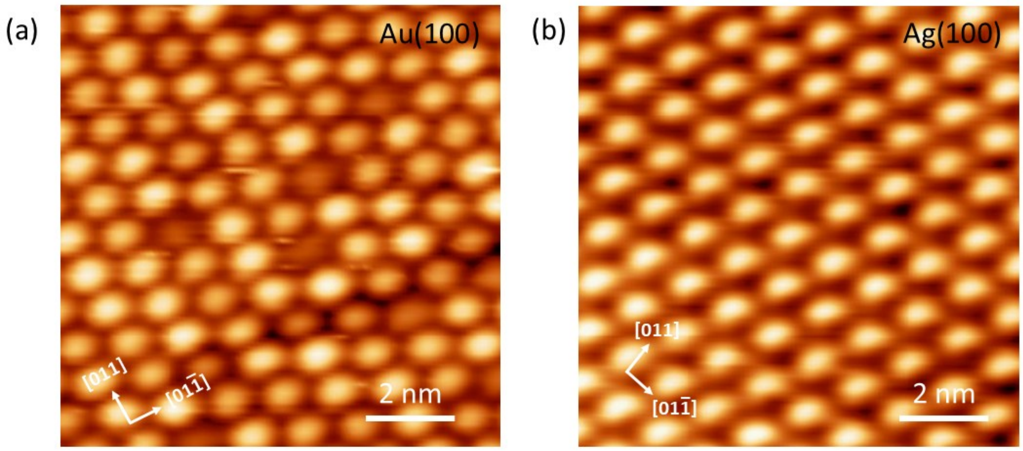

Figure S4. (a) and (b) Constant-current STM images of IPr deposition on Au(100) and Ag(100) surface at saturation coverage at room temperature (RT), respectively. STM condition (RT): (a) -2.47 V, 15 pA. (b) -2.84 V, 20 pA.

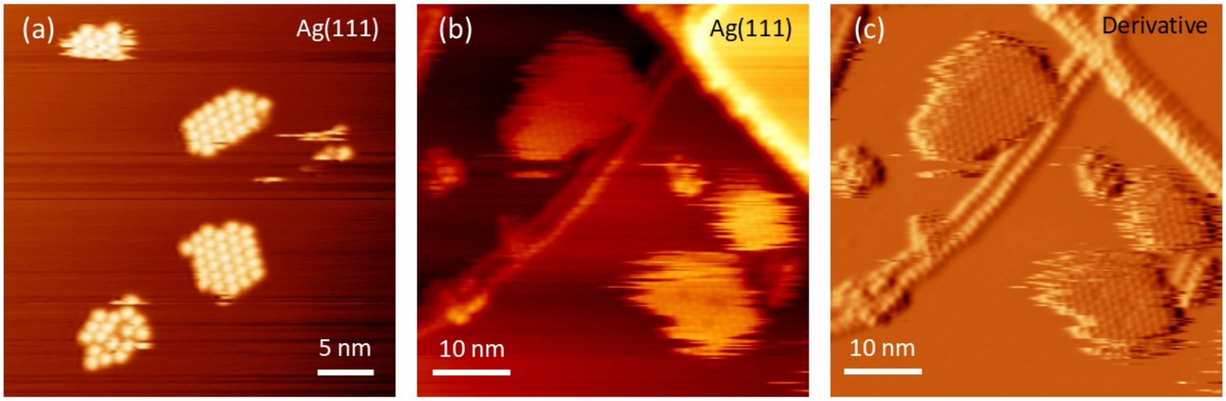

Figure S5. Constant-current STM images of sub-monolayer coverage IPr deposition on Ag(111) surface, (a) Zoom-in. (b) Zoom-out. (c) Derivative image of Figure S5b. The images consistently show the presence of self-organized assemblies at sub-monolayer coverage. STM condition (78 K): (a) -2.0 V, 30 pA. (b) and (c) -2.84 V, 60 pA.
